# Deep Learning-Based Diagnosing Structural Behavior in Dam Safety Monitoring System

**DOI:** 10.3390/s21041171

**Published:** 2021-02-07

**Authors:** Longbao Wang, Yingchi Mao, Yangkun Cheng, Yi Liu

**Affiliations:** School of Computer and Information, Hohai University, Nanjing 211100, China; wlb@hhu.edu.cn (L.W.); 191307040016@hhu.edu.cn (Y.C.); 191307040003@hhu.edu.cn (Y.L.)

**Keywords:** deep learning, node credibility, multiple relevant sequence, region evaluation, dam safety monitoring

## Abstract

Collecting a myriad of prototype data through various types of monitoring sensors plays a virtual important role in many aspects of dam safety such as real-time grasp of safety state, exposure of hidden dangers, and inspection design and construction. However, the current methods of prediction are weak in the long-term sequence of nodes with missing and abnormal error value. Moreover, the limitation caused by the apparatus, environmental factors, and network transmission can lead to the deviation and inconsistency of diagnosis and evaluation of local region. In this paper, we consider the correlation of data on nodes in the entire monitoring network. To avoid the deviation caused by noise and missing value in the single-node data sequence, we calculate the correlation between the multiple sequences. A single-node assessment model based on multiple relevant sequence (SAM) is proposed to improve the accuracy of single node assessment. Given the different nodes of a local region have varying impacts on the evaluation results, a local region evaluation algorithm based on node credibility (LREA) is presented to model the credibility of nodes in order to alleviate inconsistent evaluation results in the local region of dam. LREA can assess the dam’s operation state by considering the variations in credibility and multiple nodes coordination. The experimental results illustrate the LREA can reveal the trends of the monitoring values change in a timely and accurate way, which can elevate the accuracy of evaluation results of dam safety.

## 1. Introduction

**Figure 1:**
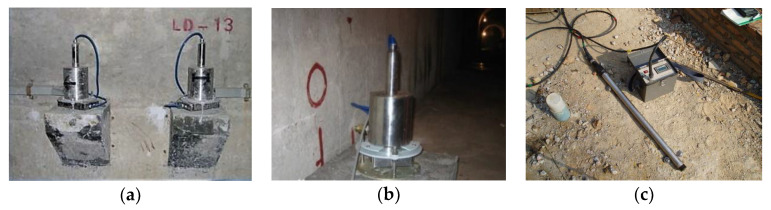
Various types of devices to measure the different physical quantities (**a**) static level; (**b**) inverted vertical meter; (**c**) seam measurement meter.

Reservoirs and dams now play a pivotal role in the regulation of the temporal and spatial distribution of water resources as well as their optimization. They also provide considerable social and economic benefits such as flood control, power generation, irrigation, water supply, transportation, and tourism. As a colossal engineering structure, dams are usually built in a strategical position yet fragile ecology, thus making their internal and external conditions very complex. To guarantee its safe operation, the following two types of measures can be adopted. (1) Engineering measures: engineering technologies are used to reinforce and maintain a dam at fixed intervals of time. (2) Non-engineering measures: these measures include flood forecasting and safety monitoring, among other means. Nowadays, various types of devices (e.g., sensor nodes) are deployed in the large dam engineering to measure the different physical quantities and sense their changes in various regions of the structure, such as deformation, stress, pressure, etc. As shown in Figure 1a–c, three types of devices are the static level, inverted vertical meter, and seam measurement meter, respectively. They are deployed to measure the deformation, stress, and pressure of the dam. 

Collecting a large amount of data through various types of monitoring sensors plays an important role in many aspects of dam safety such as real-time grasp of safety state, exposure of hidden dangers, and inspection design and construction. Therefore, in addition to diagnosing and evaluating the working behavior of dam, it is also important to accurately analyze the data from their safety nodes. 

At present, the dam safety evaluation is mainly based on the periodic post-evaluation. After a dam operates for some time (a week, a month, a quarter, a year, or a periodic inspection), the monitoring data of the dam’s operating status during this period will be collated and examined [1,2,3,4]. For the crucial nodes of a key dam region which fit under different categories of monitoring items such as deformation, seepage-pressure, stress-strain, and temperature, the specialized statistical analysis models will be constructed to analyze and predict the changing trend of single nodes data, and then evaluate the working conditions of the key dam region. The dam overall safety evaluation basically stays at data evaluation and trend analysis of key nodes of the local region of the dam’s local region [5].

Local regions of the dam under monitoring are mainly partitioned and formed by a spatial arch monitoring grid which is composed of longitudinal monitoring sections and horizontal monitoring sections, combining the concrete dam-specific monitoring design specifications, structural calculation results, and previous engineering experience. In the early phase, dam safety monitoring only paid attention to a small number of key nodes. At that time, the monitors classified the key monitoring data and judged whether the data was abnormal by the 3σ principle. With the continuous upgrade of monitoring technologies and means, the automatic dam monitoring systems have gone online, and many nodes got connected to them consequently. According to different types of monitored physical quantities, these monitoring systems can be divided into several categories of level-1 monitoring items, such as displacement, pressure, strain, and crack. Monitoring items are classified according to different monitoring technologies or means and then assigned to different nodes. As a result, they form a tree structure that consists of many layers with an entire dam at the top and nodes at the bottom. Experts assign weights to each layer of the structure and judge the overall operation state of the dam with these weights in a bottom-up approach. Besides, experience-driven assessment methods also include structural mechanics models, finite element models, spectrum analysis, statistical models, and wavelet analysis [6]. Furthermore, deformation monitoring data analysis turns out to be another means to diagnose the dam’s health status. According to the dam engineering specifications, the multi-scale fuzzy C-means-based data mining algorithm [7] is used to cluster the deformation monitoring data of a dam, compare and analyze the differences between its conventional deformation distribution diagram and deformation clustering diagram, and ultimately diagnose the operation state of the dam. The dam monitoring data contains a variety of information on effect quantity. Based on the Dempster-Shafer (DS) theory of evidence, a multi-effect quantities fusion reasoning model for dam behavior [8] is built to get the dam operation characteristics’ reasoning results. Although fitting single short-time sequence well, these traditional methods fit single short-time sequence well, these traditional methods are weak in predicting the long-term sequence of the key nodes with missing or abnormal error values, especially those playing a critical role in dam safety evaluation.

Moreover, due to the limitations of equipment, environment, network transmission and other factors, the comprehensive diagnostic evaluation of nodes deployed in the same local area is prone to deviations, resulting in inconsistent diagnostic evaluation results. Because of the above-mentioned deficiencies existing in the safety assessment of a dam monitoring region, to avoid the deviation caused by noise and missing value in the single-node data sequence, a single-node assessment model based on multiple relevant sequence (SAM) is proposed to improve the accuracy of single node assessment. To alleviate inconsistent evaluation result of the nodes in a same local region, a local region evaluation algorithm based on node credibility (LREA) is proposed to model the credibility of the nodes and achieve consistent local region evaluation results by cooperating with multiple nodes.

The rest of this paper is organized as follows. We introduce the related work in Section 2. The preliminary and problem statement are presented in Section 3. In Section 4, we present a local region evaluation algorithm based on node credibility for structural behavior diagnosing. Finally, we evaluate the performance of the proposed SAM and LREA with large-scale real dam monitoring data in Section 5, and conclude the work in Section 6.

## 2. Related Work

The dam’s local region is deployed with various types of nodes to monitor deformation, seepage-pressure, stress–strain, and temperature, among other dam engineering aspects. Therefore, monitoring data gathered from nodes can assess the local region’s operation state where such nodes are deployed. To be specific, the nodes monitor the dam working condition effect quantity of the place where it is arranged, and the patterns of how time-sequence data of nodes change to reflect the operation state of the corresponding region. With the actual monitoring data from single nodes analyzed, one can learn how the nodes are deployed separately. Next, multiple nodes can be cooperated to assess the local region’s operating state together.

### 2.1. Analysis of Single Node Values

The analysis of single-node monitoring data primarily predicts the time-sequence data of nodes, analyzes the differences between the measured (real) values and the predicted values, and identifies the specific physical characteristics changes in the local regions. The existing single-node prediction methods can be roughly divided into regression analysis methods (such as partial least squares regression method, ridge regression method, principal component regression method, and Lasso regression), time-sequence analysis (stationary time sequence [9], autoregressive-moving average (ARMA) model [10] and autoregressive integrated moving average (ARIMA) model [11], artificial neural network method, spectrum analysis method, Kalman filter method [12,13], and gray theory analysis method. Although fitting single short-time sequence well, these traditional methods are weak in predicting the long-time sequence with missing values and abnormal error values. With the development and prevalence of machine learning/deep learning in recent years, processing and predicting time sequence have increasingly been used by these following methods: Kernel Method [14,15], Online Learning [16], Support Vector Machine [17], Recurrent Neural Network (RNN) [18], and Long Short-Term Memory (LSTM) [19,20]. Both RNN and LSTM can remember the historical state of time sequence. In comparison, LSTM adds forgetting gate, input gate, and output gate to the RNN structure, which effectively prevents the gradients from vanishing or exploding, a problem that exists in RNN. Although LSTM demonstrates impressive learning ability in processing the single time sequence with long cycles and large fluctuations, it sees its ultimate prediction effects severely impacted by the non-smooth, unstable time sequence. To address the prediction and assessment errors caused by noisy or missing values in the time sequence of single nodes, we compute the node sequence correlation and harnesses the multi-correlation sequence for raising the accuracy of single node assessment.

### 2.2. Collaborative Diagnosis with Multiple Nodes

As to the evaluation of a region, many existing methods are used along with the assessment results of multiple nodes to conclude how the local region works. Hierarchical Aggregate Classification (HAC) [21] is a method used to construct a hierarchical tree of nodes: focal points can aggregate all nodes’ classification results to achieve the overall assessment of the entire network of sensors. The majority node voting scheme [22] harnesses the cluster head nodes to make statistics of node classification results in the cluster to identify abnormal states in the cluster. In these methods, the classification results of majority nodes represent those of focal points or clusters. Enhanced Naive Bayes Classifier (ENBC) [23] resorts to unsupervised learning to improve the probability distribution of multi-node classification results, thereby increasing the accuracy of event classification in the network of sensors. Majority of Methods Voting Scheme (MMVS) [24,25] integrates multiple methods and models, usually outperforming single methods. Because of the greater model complexity, ENBC and MMVS both can increase the accuracy of multi-node classification. The current methods primarily rely on most nodes or the increased model complexity to enhance the accuracy of collaborative multi-node classification single-node classification result’s credibility without considering the single-node classification result’s credibility. However, because of reasons such as apparatus, environmental factors, and network transmission, the evaluation results of different nodes in the same local region are inconsistent. Under this circumstance, treating all nodes with no distinction will lead to deviations. Therefore, we introduce a node credibility model, which describes how differently each node affects the overall evaluation results of a local region and uses the variances in credibility to unify the local region’s evaluation results produced by different nodes.

## 3. System Model and Problem Formulation

### 3.1. System Model

Regional partition algorithm (RPA) divides a structure into several single region according to the spatial-temporal characteristics of nodes, since the evaluation result of the physical state of the single region can showcase the local operation characteristics of the structure [26]. In a single region, many types of physical quantities can be observed from multiple nodes. According to the data gathered from single nodes, the changing trend of a certain physical quantity in the region can be analyzed. For example, when a single region’s stress value changes suddenly, the stress sensors will gather the changed data. Simultaneously, there is a correlation between any two changes in various types of physical quantities in a single region. Sudden changes in stress values will lead to sharp fluctuations in displacement values. Whether changes to physical quantities are abnormal can be determined regarding the time sequence revealed by nodes’ data. Therefore, we can predict the time sequence of single nodes and then compare the differences in the measured and predicted values to assess the state of physical quantities observed at single nodes. The spatial and temporal characteristics of nodes within a single region are highly correlated, which renders the time sequence of nodes highly relevant. In practical application, single nodes’ sequence data are generally characterized by being non-smooth, unstable, with noisy and missing values, thus interfering with the prediction and assessment results of single nodes. Data changes at a single node are often accompanied by synchronous data changes across multiple correlation nodes. At this moment, if multiple related node sequences are used to track and predict the data of a single node collaboratively, it is expected to deliver a better assessment performance.

A single node’s assessment results can be deemed a reflection of how the local region operates on a certain physical quantity. In this sense, it is feasible to coordinate all the nodes to evaluate a single local region’s physical state. However, because of apparatus failure, external environment, and other factors, the monitoring sequence data from a single node are not reliable, so different nodes exert various impacts on the overall region’s overall evaluation. In fact, multiple sequences can be used to improve the single-node assessment results’ credibility. However, suppose multiple highly correlated time sequence all become abnormal. In that case, there will be a situation where “two negatives make a positive,” that is, multiple abnormal time sequences are all regarded as normal ones, which leads to deviations in the single-region evaluation results. To boost the accuracy of single-region evaluation results, the credibility of single-node is modeled so that the credibility can be used to describe the impact of single nodes on the single-region evaluation results. Based on the credibility, the assessment results of different nodes will then be coordinated and integrated so that a single region can be evaluated more accurately by distancing from the nodes with low credibility and getting close to those with high credibility wherever possible.

### 3.2. Problem Formulation

**Figure 2:**
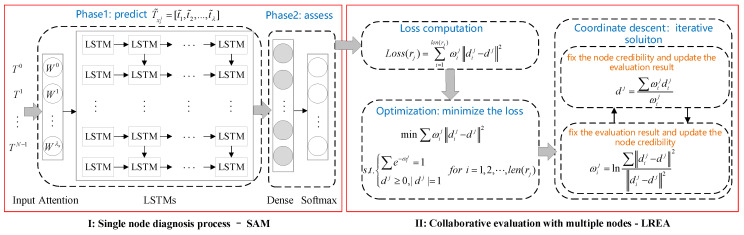
Evaluation procedure of a local region.

The local region assessment includes two phases: First, predict the time sequence of a single node and then use the differences between the predicted values and the real values to assess the operation state of the node; second, coordinate multiple nodes to assess the operation state of the corresponding local region. The detailed process of which region $r_{j}$ is evaluated at the time point *T* is elaborated in Figure 2.

(1) Single node diagnosis process

During the process of single node diagnosis, we predict and assess the node $x_{i}^{j}$ with the data of historical time sequence. At the time point *T*, select the historical sequence $T_{x_{i}^{j}}=\left[t_{1},t_{2},\cdots ,t_{\lambda }\right]$ with the length of λ to train and learn about how to map the single-node prediction model:

(1) $$predict(x_{i}^{j}):T_{x_{i}^{j}}\to t_{T}$$

According to the single node prediction model, the node $x_{i}^{j}$ sees its predicted value at the time point *T* computed as $t_{T}$, and meanwhile every historical moment before the time point *T* has a predicted value calculated as ${\tilde{T}}_{x_{i}^{j}}=\left[{\tilde{t}}_{1},{\tilde{t}}_{2},\cdots ,{\tilde{t}}_{\lambda }\right]$. The real value $T_{x_{i}^{j}}$ and the predicted value ${\tilde{T}}_{x_{i}^{j}}$ are combined to assess the operation state of the node:

(2) $$assess(x_{i}^{j}):\{T_{x_{i}^{j}},{\tilde{T}}_{x_{i}^{j}}\}\to d_{i}^{j}$$

where $d_{i}^{j}$ is the assessment result of a single node as a probability vector. For instance, the node $x_{2}^{1}$ in the region $r_{1}$ may get its operation state assessed as any of the following preset results: “Good,” “Normal,” “Checked,” “Abnormal.” After prediction and assessment, it is obtained that $d_{2}^{1}=\left[0.65,0.2,0.1,0.05\right]$, which means the above four operation states correspond to the probabilities of 65%, 20%, 10%, and 5%, respectively. With the maximum probability adopted, the node $x_{2}^{1}$ has its operation state assessed as “Good.”

(2) Collaborative evaluation with multiple nodes

For the collaborative evaluation with multiple nodes, we predict and assess each node in a local region to obtain $\left\{d_{i}^{j}|i=1,\cdots ,len(r_{j})\right\}$. It integrates the operation states of multiple nodes to assess how the corresponding region operates. The evaluation process of a local region can be defined as below:

(3) $$eval(r_{j}):\{d_{i}^{j}|i=1,\cdots ,len(r_{j})\}\to d^{j}$$

where $d^{j}$ indicates the consistent evaluation result of the region reached by all nodes available. For instance, suppose the region $r_{1}$ takes the assessment result as its operation state, all nodes available at the local region are coordinated to produce the evaluation result $d^{1}=\left[0.8,0.1,0.09,0.01\right]$, which means the region is assessed as “Good” (with a probability of 80%).

## 4. Local Region Evaluation Based on Node Credibility

### 4.1. Single Node Diagnosis

**Figure 3:**
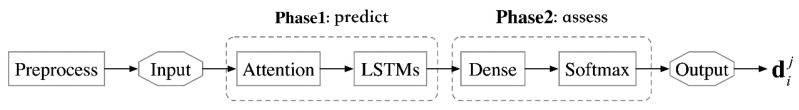
Single-node assessment model based on the collaboration of multiple nodes.

Given the fact that the data on a time sequence of single nodes are non-smooth, unstable, with noisy and missing values in actual operation, we propose the single-node assessment model based on multiple relevant sequence (SAM). Within SAM, the data on the time sequence of nodes are pre-processed and normalized, so that cosine similarity can be used to compute the relevancy of the sequence; select multiple correlation sequence as inputs and add the attention layer to assign multiple sequences with attention weights; adopt the LSTM layer to make a prediction and complete the assessment of a single node through the Softmax layer. The structure of the SAM is shown in Figure 3. The single-node evaluation process includes two stages.

(1) Pre-process the time sequence of a node, calculate the correlation coefficients between the node and other ones in time sequence as inputs, and harness the attention layer and LSTMs to finish the single-node prediction based on multiple correlation sequence.
(2) Based on the sequence of real and predicted values produced by a node, $d_{i}^{j}$ can be obtained by assessing the state of physical quantities observed at the single node $x_{i}^{j}$ through the Dense layer and the Softmax layer.

#### 4.1.1. Time Sequence Normalization

**Figure 4:**
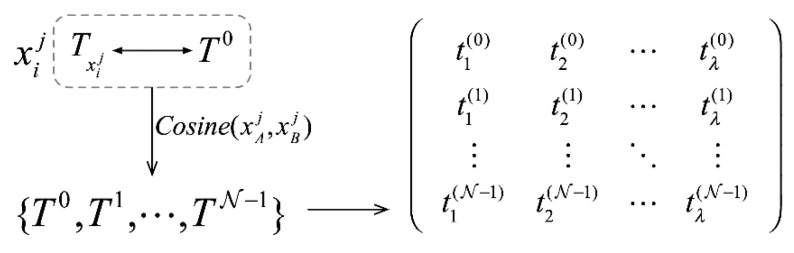
Node sequence pre-processing.

In a local region, various nodes are deployed to oversee its different physical quantities. As a result, related data come in varying units and ranges. Before single nodes are predicted and assessed, they should be normalized. The node $x_{i}^{j}$ sees its original time sequence $T_{x_{i}^{j}}=\left[t_{1},t_{2},\cdots ,t_{\lambda }\right]$ normalized with the following equation:

(4) $$t_{i}=\frac{t_{i}-\mu_{T_{x_{i}^{j}}}}{\sigma_{T_{x_{i}^{j}}}}$$

where $\mu_{T_{x_{i}^{j}}}$ and $\sigma_{T_{x_{i}^{j}}}$ represent the mean value and standard deviation of the sequence, respectively. Any null value (NULL, NAN) existing in the sequence will be set as “0.” Then compute the correlation sequence of $T_{x_{i}^{j}}$ and construct SAM inputs, with the process seen in detail as shown in Figure 4.

When data are normally distributed or undergo standardized processing, Pearson correlation coefficient, cosine similarity, and squared Euclidean distance can be considered as equivalent. For the sake of calculation convenience, cosine similarity is used to compute the correlation between the two nodes $x_{A}^{j}$ and $x_{B}^{j}$:

(5) $$Cosine(x_{A}^{j},x_{B}^{j})=\frac{T_{x_{A}^{j}}\cdot T_{x_{B}^{j}}}{\left\|T_{x_{A}^{j}}\|\times \|T_{x_{B}^{j}}\right\|}$$

To compute the correlation coefficients between all the nodes available in a local region, we need to select the sequence $\left\{T^{0},T^{1},\cdots ,T^{N-1}\right\}$, consisting of N−1 nodes featuring the highest correlation with the sequence $x_{i}^{j}$ (denoting $T_{x_{i}^{j}}$ as $T^{0}$). In the sequence $\left\{T^{0},T^{1},\cdots ,T^{N-1}\right\}$, N should be a natural number equal to or greater than “1.” When N is equal to “1,” the sequence of $x_{i}^{j}$ itself will be selected. If N is equal to “3,” it means two correlation sequence should be selected. The selection of N value will be verified in the following experiments.

#### 4.1.2. Single Node Diagnosis Model

**Figure 5:**
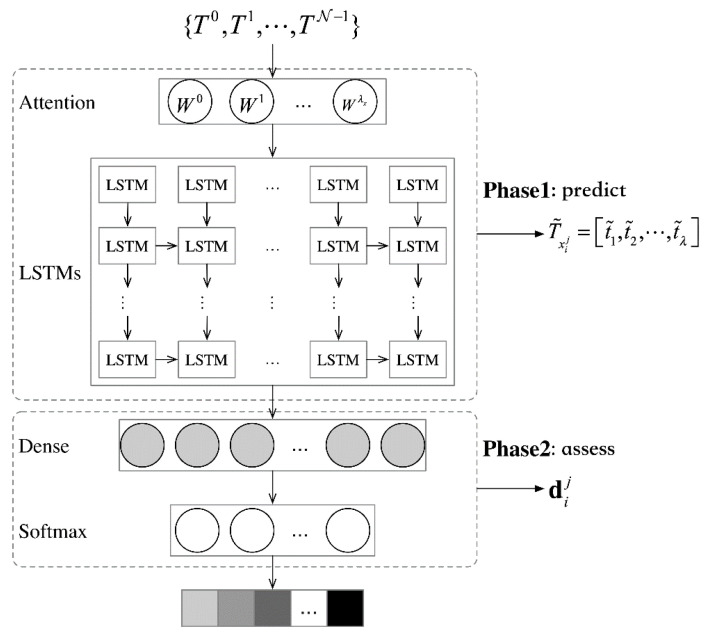
The diagnosis model of single node.

After the sequence of nodes is pre-processed, we can compute the correlation of multiple sequences from different nodes $\left\{T^{0},T^{1},\cdots ,T^{N-1}\right\}$, which is as the input of diagnosis model. The diagnosis of single nodes is based on the multiple nodes with the spatial-temporal correlation, the specific procedure can be seen in Figure 5.

(1) Prediction of single node. A related prediction is completed by using the attention layer and the LSTM layer. Considering that each sequence’s input bears varying magnitudes of importance in the prediction process, the attention layer is employed to assign each sequence with a weight: $\left\{W^{0},W^{1},\cdots ,W^{N-1}\right\}$. With the attention weights applied to the inputted sequence, we can obtain $\left\{W^{0}T^{0},W^{1}T^{1},\cdots ,W^{N-1}T^{N-1}\right\}$ as the inputs on the LSTM layer. Weights are updated through error backpropagation. Predictions on the LSTM layer led to the predicted value ${\tilde{T}}_{x_{i}^{j}}$ of the $x_{i}^{j}$ sequence. For the node $x_{i}^{j}$, its prediction error can be obtained through the root-mean-square deviation between the original time sequence $T_{x_{i}^{j}}$ and the predicted value of such sequence ${\tilde{T}}_{x_{i}^{j}}$.
  (6) $$RMSE=\sqrt{\|T_{x_{i}^{j}}-{\tilde{T}}_{x_{i}^{j}}{\|}^{2}}$$
(2) Diagnosis of single node. For the node $x_{i}^{j}$, based on the real value $T_{x_{i}^{j}}$ and the predicted value ${\tilde{T}}_{x_{i}^{j}}$, its assessment result $d_{i}^{j}$ can be obtained through the Dense and Softmax layers. Cross entropy (CE) is used as an assessment error. A SAM error is composed of prediction error and assessment error, with the loss function displayed as below. A composition error $Loss_{SAM}$ can alleviate the situation where a single error (RMSE or CE) is optimized excessively, hereby protecting the model from over-fitting.
  (7) $$Loss_{SAM}=RMSE+CE$$

### 4.2. Local Region Evaluation Model with Multiple Nodes

**Figure 6:**
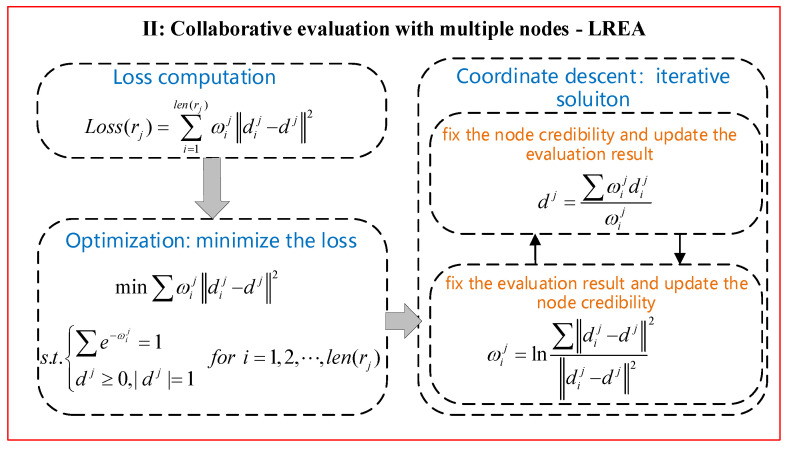
Collative evaluation procedure with multiple nodes.

It is possible to assess all nodes in the local region by prediction, diagnosis and then integrate these nodes’ assessment results to evaluate how their corresponding regions operate. However, different single nodes may be varying in credibility, due to factors such as network transmission, environmental impact, and nodes themselves. If all nodes are treated with no distinction, it will obtain the evaluation results that deviate from actuality through integrating the assessment results of all nodes. To obtain more accurate evaluation results for a local region, a local region evaluation algorithm based on node credibility (LREA) was proposed. Note that different nodes in the same local region have inconsistent evaluation results and different nodes’ credibility. Therefore, the local region evaluation is converted into a matter about how to optimize the node credibility and assessment results. On that premise, the coordinate descent method can be used to calculate a local region evaluation result. The processing flow diagram is shown in Figure 6.

#### 4.2.1. Node Credibility

How creditable the node $x_{i}^{j}$ take an effect on the evaluation of the local region $r_{j}$ is defined as the credibility $\omega_{i}^{j}=\{x_{i}^{j},r_{j}\}$ in a bid to measure the degree of influence which the node will exert on the local region. In physical terms, credibility $\omega_{i}^{j}$ denotes the role that the physical quantities monitored by the node $x_{i}^{j}$ play in the evaluation of the local region $r_{j}$. Suppose the middle part of an arch dam is designated as the local region under monitoring and that the most obvious deformation and displacement are identified from such part, the nodes deployed for monitoring deformation and displacement will take a bigger part in the local region evaluation than other nodes. On the other hand, the credibility $\omega_{i}^{j}$ can also indicate the difference between the single node assessment result $d_{i}^{j}$ and the local region evaluation result $d^{j}$. The higher $\omega_{i}^{j}$ is, the nearer $d_{i}^{j}$ and $d^{j}$ tend to be. Apparently, the local region evaluation result $d^{j}$ will approach the assessment results produced by most highly creditable nodes.

All nodes available in the same local region should see their credibility subject to a certain type of constraint to facilitate comparison and calculation. An intuitive choice will be $\sum \omega_{i}^{j}=1,\;\omega_{i}^{j}\in [0,1]$, where all nodes have their credibility be non-negative and added up to “1.” Below are some problems that may arise from there.

(1) When $\omega_{i^{\prime }}^{j}\gg \max\{\omega_{i}^{j}|i=1,\cdots ,len(r_{j});i\neq i^{\prime }\}$ happens, it means that a certain node has its credibility far greater than that of any remaining node. For example, a certain node’s credibility is “1” and that of any other node is “0.” The assessment result of the node will directly restrain that of any other node, leading to $d^{j}\approx d_{i}^{j}$, that is, a locally optimal solution.
(2) $\omega_{i}^{j}\in [0,1]$ means a decimal. When there are many nodes, some nodes are likely to have their credibility $\omega_{i^{\prime }}^{j}\approx 0$, which results in more errors or an error of division by 0 from the calculation process.
(3) The function f(x)=x considers its derived function as a constant, a situation defying the solution with gradients.

To avoid the above problems, $\sum e^{-\omega_{i}^{j}}=1$ is selected as the credibility constraint. In the function $g(x)=e^{-x}$, its derived function is −g(x), a design conducive to obtaining the closed-form solution. In the case of special values and boundary values, we can set $g(0)=1,g(1)=e^{-1},g(\infty )=0$, to avoid the occurrence of calculation error.

At the beginning, the node credibility was initialized randomly and got constantly corrected with the measured data. The node time sequence data in dynamic changes make it possible for the node credibility distribution to match up to the actual operation state of the local region under monitoring. On this basis, credibility can be used to coordinate multiple nodes for local region evaluation.

#### 4.2.2. Region Evaluation

Define the single node assessment result of the single-node $x_{i}^{j}$ as $d_{i}^{j}$ and integrate multiple nodes to get the local region evaluated as $d^{j}$. Given that different nodes vary in credibility, they will affect the local region evaluation result to different extents. As to the final revaluation result, it can be felt that the higher node credibility leads to the smaller evaluation error, and the lower node credibility leads to greater evaluation error. The local region evaluation error is defined as below:

(8) $$Loss(r_{j})=\sum_{i=1}^{len(r_{j})}\omega_{i}^{j}∥d_{i}^{j}-d^{j}{∥}^{2}$$

where $Loss(r_{j})$ is the sum of all node assessment errors, and $len(r_{j})$ is the total number of nodes. In Equation (8), $\omega_{i}^{j}∥d_{i}^{j}-d^{j}{∥}^{2}$ denotes the assessment error of the single node $x_{i}^{j}$, and $\omega_{i}^{j}$ means the credibility of the node $x_{i}^{j}$. When $\omega_{i}^{j}$ is very large and the difference between $d_{i}^{j}$ and $d^{j}$ is considerable, $\omega_{i}^{j}∥d_{i}^{j}-d^{j}{∥}^{2}$ will become quite large. By the same token, when $\omega_{i}^{j}$ is quite small yet $∥d_{i}^{j}-d^{j}{∥}^{2}$ turns out quite large, $\omega_{i}^{j}∥d_{i}^{j}-d^{j}{∥}^{2}$ will take on a relatively smaller value.

Therefore, minimizing $Loss(r_{j})$ keeps the local region evaluation result $d^{j}$ away from the highly creditable nodes (when $\omega_{i}^{j}$ is relatively large) yet close to the poorly creditable nodes (when $\omega_{i}^{j}$ is relatively small).

Now, considering the node credibility constraints and assessment errors, the local region evaluation process is converted into the optimization solution shown as below:

(9) $$Loss(r_{j})=\sum_{i=1}^{len(r_{j})}\omega_{i}^{j}∥d_{i}^{j}-d^{j}{∥}^{2}$$

The objective of optimization is to minimize the local region evaluation error $Loss(r_{j})$. The constraints conditions include $\sum e^{-\omega_{i}^{j}}=1$ and $d_{i}^{j}\geq 0,|d_{i}^{j}|=1$. The former condition is used to restrain the node credibility, while the latter one is intended to make sure the local region evaluation result $d^{j}$ will not be negative and that all component probabilities can be added up to “1.”

The optimization problem illustrated in Equation (9) tries to search in the solution space for the node credibility $\omega^{j}=\{\omega_{i}^{0},\omega_{i}^{1},\cdots ,\omega_{i}^{len(r_{j})}\}$ and the local region evaluation result $d^{j}$. This is a non-convex optimization problem to which the gradient method is not applied. Given this, the coordinate descent method is adopted for a solution. At each iteration, the search is carried out along one direction in which the optimization problem is solved to update the node credibility and the local region evaluation result alternately.

### 4.3. Iterative Solution

The process of the iterative solution with the coordinate descent method takes place in two steps. Step 1: fix the node credibility and update the local region evaluation result. Step 2: fix the local region evaluation result and update the node credibility. Each round of iteration pushes the optimization problem closer to the optimal solution. The above two steps need to be executed alternately until the final convergence is realized.

(1) Update the revaluation results

When any node $x_{i}^{j}$ gets its credibility $\omega_{i}^{j}$ fixed, updating the single region evaluation result $d^{j}$ can minimize the overall evaluation error. Since $\omega_{i}^{j}$ is supposed as known, the partial derivative of $Loss(r_{j})$ to $d^{j}$ is calculated directly as below:

(10) $$\frac{\partial Loss(r_{j})}{\partial d^{j}}\propto \sum \omega_{i}^{j}(d_{i}^{j}-d^{j})$$

Suppose the partial derivative of Equation (10) as “0,” it can be solved as below:

(11) $$d^{j}=\frac{\sum \omega_{i}^{j}d_{i}^{j}}{\sum \omega_{i}^{j}}$$

It should be noted that $\sum \omega_{i}^{j}\neq 1$ (the node credibility restraint is $\sum e^{-\omega_{i}^{j}}=1$). Besides, since the single node assessment result can meet the constraint condition $d_{i}^{j}\geq 0,|d_{i}^{j}|=1$, the single region evaluation result solved by Equation (11) is bound to satisfy the above restraint condition.

(2) Update the credibility of nodes

Next, we will fix the single region evaluation result $d^{j}$ and update the node credibility $\omega_{i}^{j}$. The Lagrange method is adopted to rewrite the optimization objective function into Equation (12):

(12) $$L(\alpha ,d^{j})=\sum \omega_{i}^{j}∥d_{i}^{j}-d^{j}{∥}^{2}+\alpha (\sum e^{-\omega_{i}^{j}}-1)$$

where α is the Lagrange multiplier factor. The partial derivative is solved to $\omega_{i}^{j}$ as below:

(13) $$\frac{\partial L(\alpha ,d^{j})}{\partial \omega_{i}^{j}}=∥d_{i}^{j}-d^{j}{∥}^{2}-\alpha e^{-\omega_{i}^{j}}$$

Suppose Equation (13) is equal to “0,” it can be solved as below:

(14) $$e^{-\omega_{i}^{j}}=\frac{∥d_{i}^{j}-d^{j}{∥}^{2}}{\alpha }$$

With the node credibility constraints, we sum the two sides of Equation (14) as $\sum e^{-\omega_{i}^{j}}=\sum \frac{∥d_{i}^{j}-d^{j}{∥}^{2}}{\alpha }=1$, and solve the Lagrange multiplier factor $\alpha =\sum ∥d_{i}^{j}-d^{j}{∥}^{2}$ which is substituted into Equation (14) as follows:

(15) $$L(\alpha ,d^{j})=\sum \omega_{i}^{j}∥d_{i}^{j}-d^{j}{∥}^{2}+\alpha (\sum e^{-\omega_{i}^{j}}-1)$$

Equations (11) and (15) are adopted to update the single region evaluation result $d^{j}$ and the node credibility $\omega_{i}^{j}$ alternately.

### 4.4. Local Region Evaluaiton Algorithm Based on Node Credibility

To address the inconsistent evaluation results of a local region that arise from the varying levels of node credibility, the paper proposes a local region evaluation algorithm based on node credibility (LREA, or Algorithm 1 shown as below). LREA receives the assessment results of multiple nodes within a single region $\left\{d_{i}^{j}|i=1,2,\cdots ,len(r_{j})\right\}$ and the iteration error threshold ϵ as inputs and meanwhile works out the single region evaluation result $d^{j}$ and the node credibility $\omega^{j}$ as outputs.
**Algorithm 1** Local region evaluation algorithm based on node credibility (LREA)**Input**: multiple node assessment results $\left\{d_{i}^{j}|i=1,2,\cdots ,len(r_{j})\right\}$, the iteration error threshold ϵ **Output:** single region evaluation result $d^{j}$, node credibility $\omega^{j}$1: INITIALIZE $\omega^{j}$, $d^{j}(0)$, $d^{j}(1)$//Initialize node credibility and the single region evaluation result2: t=1           //Rounds of iteration3: WHILE $\sqrt{\|d^{j}(t)-d^{j}(t-1){\|}^{2}}\geq \epsilon$  //Compute and define iteration error4:    $d^{j}(t+1)=\sum \omega_{i}^{j}(t)d_{i}^{j}/\sum \omega_{i}^{j}(t)$  //Update single region evaluation result5:    $\omega_{i}^{j}(t+1)=\ln\left(\sum ∥d_{i}^{j}-d^{j}(t){∥}^{2}/∥d_{i}^{j}-d^{j}(t){∥}^{2}\right)$  //Update node credibility6:    t=t+1
7:    $\omega^{j}=\{\omega_{i}^{0}(t),\omega_{i}^{1}(t),\cdots ,\omega_{i}^{len(r_{j})}(t)\}$  //Save node credibility8:    $d^{j}=d^{j}(t)$   //Save single region evaluation result 9: END WHILE10: Return $\omega^{j}$, $d^{j}$


In Algorithm 1, the node credibility $\omega^{j}$ is initialized using the historical credibility, and the single region evaluation results (initial) $d^{j}(0)$ and $d^{j}(1)$ are initialized randomly. $d^{j}(1)$ and $\omega_{i}^{j}(t)$ represent the single region evaluation result in the *t* round of iteration and the value of node credibility, respectively. Iteration error is computed using the single region evaluation results in two rounds of iteration $d^{j}(t)$ and $d^{j}(t-1)$. Then $d^{j}$ and $\omega^{j}$ are updated alternately as instructed by Equations (11) and (15). LREA keeps converging until the iteration error threshold ϵ is reached. After that, the algorithm will return the single region evaluation result and node credibility.

## 5. Performance Evaluation

### 5.1. Experiments Setup

#### 5.1.1. Datasets

In order to show that the proposed the region evaluation based on node credibility (LERA) works well with various kinds of datasets, we have chosen the real dam safety monitoring dataset. The real dataset from the highest arch dam in the world is from 01 January 2017 to 31 December 2017 of 964 sensor nodes, which has 350,000 data items recording the sensors’ types, the spatial coordination positions, time slots, and the observed data. According to the design specification of dam safety monitoring systems, the dam is divided into 34 areas, which are distributed as shown in Figure 5. When the existing models are used to predict the displacement change of Node P04618 in the dam safety monitoring system, the predicted values often tend to be volatile at a pace lagging the real values. Therefore, Node P04618 is selected as an object of a single node prediction and assessment experiment to assess the performance of SAM. Multiple nodes are coordinated to evaluate the local region $r_{1}$ collaboratively and to analyze the performance of LREA.

**Figure 7:**
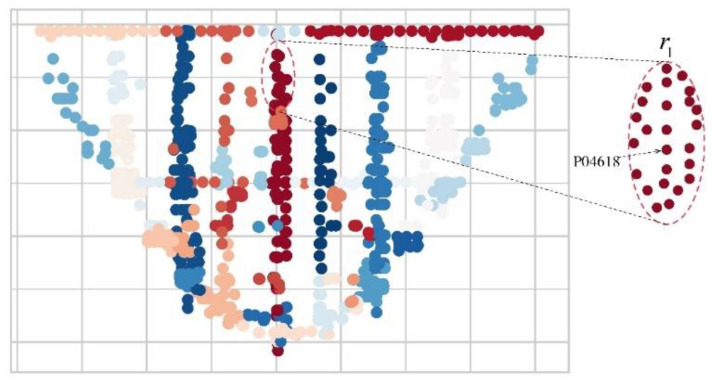
All local regions and local region $r_{1}$.

Within the set of data measured from 1 January 2017 to 31 December 2017 the single local region $r_{1}$ is chosen from the current 34 local regions of the dam under monitoring for assessment as shown in Figure 7. The local region $r_{1}$, located in the middle part of the dam, consists of 31 nodes. According to the design specifications and engineering experience in dam safety monitoring, the 31 nodes are grouped into 11 categories (C1–C11). Under the same category are highly similar nodes, of which a Key Node is selected to represent the entire category. Key Nodes map on to categories as shown in Table 1. Take Category C1, for example. There are three nodes under C1, that are, P04616, P04617, and P04618, of which P04618 is chosen as the Key Node. The experimental results show that the 11 Key Nodes produce the assessment results the same as those of the 31 nodes. Given this, the 11 Key Nodes are used to display the engineering instance as below.

**Figure 8:**
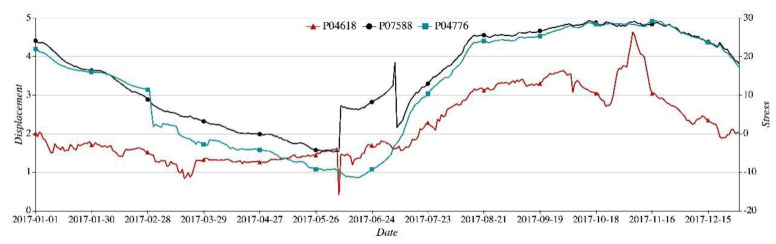
Time sequence of key nodes P04618, P07588, and P04776.

The dam is a type of double-curvature arch dam. The local region $r_{1}$, located in the upper part of the crown cantilever, comes in a complicated force-bearing structure, thus triggering the most significant displacement on a cumulative basis. Take the key nodes P04618 (displacement), P04776 (stress), and P07588 (stress) for example. The three key nodes each presented in 2017 a time sequence curve as shown in Figure 8. The three curves are vastly different in amplitude of variation and volatility.

Options for a single node assessment result or a single region evaluation result may be “Good,” “Normal,” “Checked,” or “Abnormal” (which correspond to 0, 1, 2, and 3, respectively). Key Nodes are predicted and assessed using SAM. Then LREA is employed to coordinate multiple nodes and evaluate a single region collaboratively. Taking the date of 12 September 2017 as an example, the single node assessment results and single region evaluation results are shown in Table 2.

In Table 2, Column 4 displays the node credibility, which meets the credibility constraint $\sum_{i=1}^{11}e^{-\omega_{i}^{1}}=1$. The single region evaluation result is $d^{1}=[0.315,0.211,0.229,0.245]$, corresponding to the operation state of “Checked” and consistent with the actual evaluation result. To be specific, the key nodes P04618, P06706, P07045, and P08421 see their operation state assessed as “2,” and the key nodes P04776, P05257, P06152, and P07857 post an assessment result of “1.” However, the former group outperforms the latter group in terms of credibility, so the single region gets its operation state assessed as “2.” Moreover, since the node P04618 is in the middle part of the single region, its displacement pattern plays a decisive role in the region’s operation state. The node bears high credibility accordingly. The node P08252 delivers the credibility of 1.770, the lowest level among all nodes. This situation can be attributed to the existence of missing or noisy data in the node sequence.

**Figure 9:**
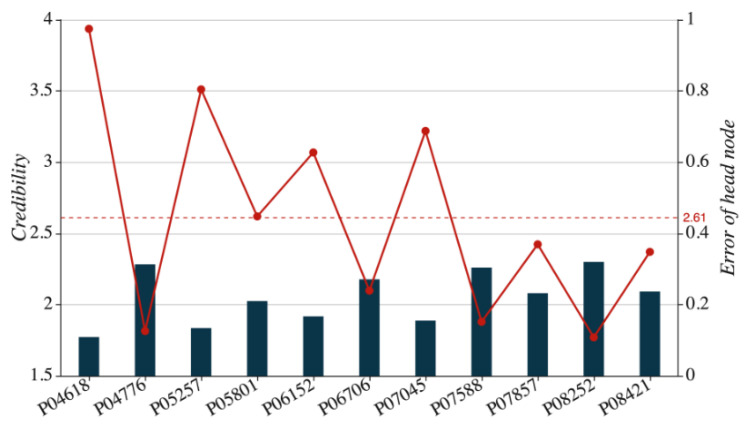
Credibility analysis of key nodes.

With the node assessment error as $err(x_{i}^{j})=\;∥d_{i}^{j}-d^{j}{∥}^{2}$, we make statistics of key node credibility and assessment error as shown in Figure 7. In the figure below, the horizontal axis indicates the serial numbers of nodes. The scatter dotted line, corresponding to the left vertical axis, tells the credibility of each node. The dotted line in parallel to the horizontal axis exhibits the average value of credibility, and the columns, along with the right vertical axis, indicate assessment errors. As shown in Figure 9, the assessment error is inversely proportional to the credibility of a node: the higher the one, the lower the other. Nodes P04618, P05257, P06152, and P07045 can be taken as examples in this case. Even though the credibility of node P06706 falls below the average level and its assessment error is quite high, it is proven that its predicted assessment result is consistent with the final evaluation result of the single region. The node’s assessment error can be corrected by other nodes with higher levels of credibility.

#### 5.1.2. Baselines

In the dam structural behavior evaluation, the commonly used single-node prediction and assessment methods or models include the auto-regressive integrated moving average model (ARIMA), support vector machine (SVM), exponential fitting, and polynomial fitting. When it comes to the dam safety operation, and maintenance practice, the dynamic optimal combined model (CM) is adopted to conduct the single-node prediction and assessment. CM represents the best prediction and assessment performance of ARIMA, SVM, and fitting models. SAM and CM will see their performance compared in a single-node prediction and assessment experiment respectively.

At present, the multiple node coordination methods are hierarchical agglomerative clustering (HAC) [21], enhanced Naive Bayes classifier (ENBC) [24], and the majority of methods voting scheme (MMVS) [25]. MMVS integrates multiple traditional methods including Bayes classifier, decision-making tree, BP neural network, support vector machine, and k-nearest neighbor. LREA, HAC, ENBC, and MMVS are compared and analyzed in a single region evaluation experiment.

#### 5.1.3. Evaluation Metrics

(1) Single node prediction experiment: SAM and CM are used to measure the daily lateral displacement of the node P04618 from 1 January 2017 to 31 December 2017. With the measured values dotted into curves, we can demonstrate how the trends of predicted and real value change and then use the root mean squared error (RMSE) to measure the accuracy and stability of related methods.
(2) Single node assessment experiment: SAM and CM are employed to assess the predicted values and true values of nodes. In the actual engineering work, nodes may see their operation state fitting under any of the following four categories of assessment results: “Good,” “Normal,” “Checked,” or “Abnormal.” Then we will make statistics of the accuracy rates of assessment results produced by SAM and CM both as a whole and on a category-specific basis. The resulting experiment results are presented in the form of a confusion matrix.
(3) Experiment on the number of correlation sequence and correlation coefficient thresholds on the SAM performance: Within SAM, multiple sequences are harnessed to make predictions and assessments. Therefore, it is necessary to verify how the number of correlation sequence and correlation coefficient thresholds impact the model’s accuracy and stability. The resulting assessment indicator is RMSE.
(4) LREA convergence analysis: The analysis is made to verify the impact of the number of nodes on the LREA convergence and the changes in iteration error.
(5) Single region evaluation experiment with such methods or models as LREA, HAC, ENBC, and MMVS: Compare and analyze the accuracy rates of local region evaluation results produced by LREA and the three other methods currently adopted.

### 5.2. Experiment Results Analysis

#### 5.2.1. Single Node Prediction Experiment

**Figure 10:**
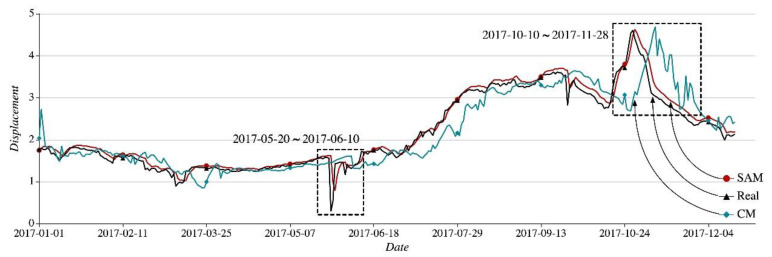
Prediction Results from 1 January 2017 to 31 December 2017.

The node P04618 of the single region $r_{1}$ sees its displacement subject to sharp fluctuations and the value changes. SAM and CM are used to predict the daily displacement of the node over the period from 1 January, 2017 to 31 December, 2017 with the experiment results shown in Figure 10. SAM, CM, and Real, along with the legends and arrows on the right corner of the figure, mean the curve of predicted values with SAM, the curve of predicted values with CM, and the curve of real values. A holistic look at the figure reveals that the SAM curve almost overlaps the Real curve, while the CM curve clearly diverges from the Real curve.

**Figure 11:**
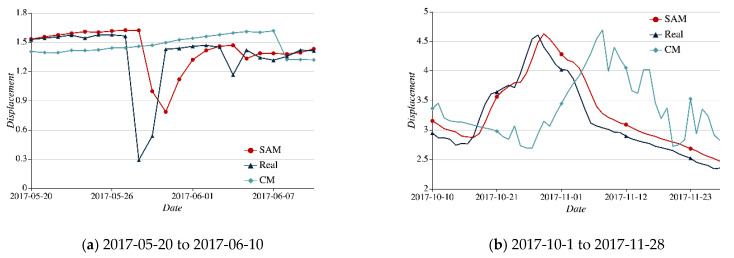
Results comparison with SAM and CM algorithms.

Taking the two periods of time marked with the dotted box, that is, 28 May 2017–4 June 2017 and 10 October 2017–28 November 2017 we find that the SAM, CM, and Real curves are divergent from each other greatly. The details can be seen in Figure 11a,b.

In Figure 11a, as the Real curve plunges on both 28 May 2017 and 4 June 2017 the CM curve takes on a trend of mild increase. However, the curve with SAM follows the Real curve to drop promptly. As shown in Figure 11b, the Real curve reaches a prominent peak from 10 October 2017 to 28 November 2017. Over the same period, the SAM curve keeps up with the Real curve in time, while the CM curve fluctuates considerably and falls far behind the pace at which the Real curve changes.

**Figure 12:**
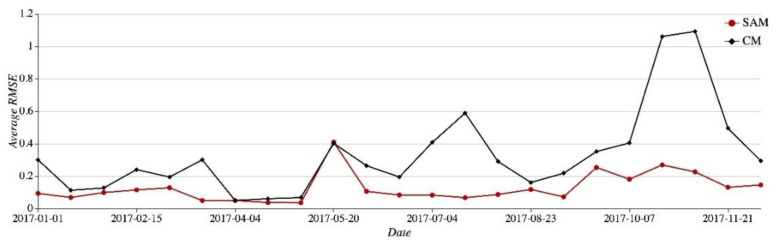
Average RMSE with SAM and CM in the 15 days interval.

SAM resorts to multiple sequences for prediction. It will not skip or fluctuate suddenly in response to a sharp change in the Real curve. Its predicted values tend to be relatively stable. However, the currently used CM is unable to reveal the changing pattern of the Real curve promptly. From 1 January 2017 to 31 December 2017 divided into many 15-day periods, we make statistics of average RMSE values produced by SAM and CM, respectively. The details can be seen in Figure 12. In the figure, the SAM RMSE curve always lies below the CM RMSE curve. Within the periods when the Real curve flattens, like the period from 16 January 2017 to 1 May 2017 the SAM RMSE curve and the CM RMSE curve are relatively close to each other. But over the period from 10 October 2017 to 28 November 2018, when the Real curve fluctuates sharply, the CM RMSE curve goes up remarkably yet the SAM RMSE curve manages to remain steady.

#### 5.2.2. Single Node Diagnosis

**Figure 13:**
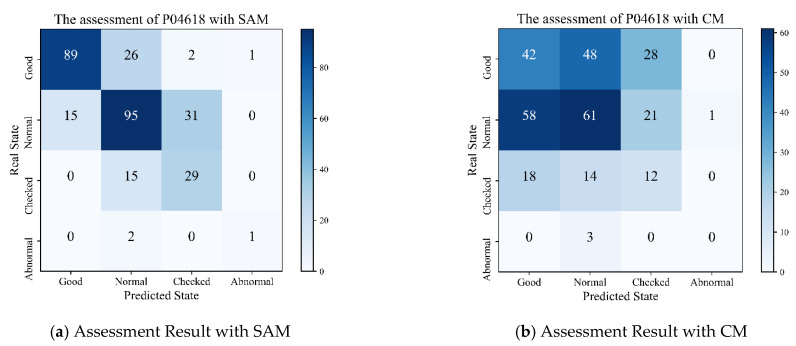
Assessment results of node P04618.

SAM and CM assess a single node’s operation state using the predicted values and real values of the node. Let us take the predicted values and real values of the node P04618 from 1 January 2017 to 28 February 2017 as a historical sample to assess the node’s operation state from 1 March 2017 to 31 December 2017 (the assessment result may be any of the following four options: “Good,” “Normal,” “Checked” or “Abnormal”). The assessment results with SAM and CM are presented in the form of a confusion matrix as Figure 13a,b. The number of days under assessment totals 306. The horizontal and vertical axes represent the predicted state and the real state, respectively.

The node P04618 sees its operation state assessed as “3” (Abnormal) throughout the year. The limited quantity leads to very poor prediction accuracy with SAM and CM, so we do not analyze the abnormal state. A diagonal line in a confusion matrix means the consistency between the predicted state and the real state. While analyzing the remaining three states (“Good,” “Normal,” and “Checked”), we find that their assessment accuracy rates with SAM stand at 0.75, 0.67, and 0.66, respectively, while those rates with CM come at 0.36, 0.43, and 0.27. It can be concluded that SAM outperforms CM, concerning the accuracy rate of any of the three states. The overall assessment with SAM and CM registers an accuracy rate of 70.0% and 37.6%, respectively. There is a huge gap of 32.3% between the two. Besides, SAM makes less error in the assessment of each state than CM, which lends SAM a more leading edge over the latter in overall assessment accuracy.

#### 5.2.3. Impact on the SAM Performance

(1) The Number of Correlation Sequence

**Figure 14:**
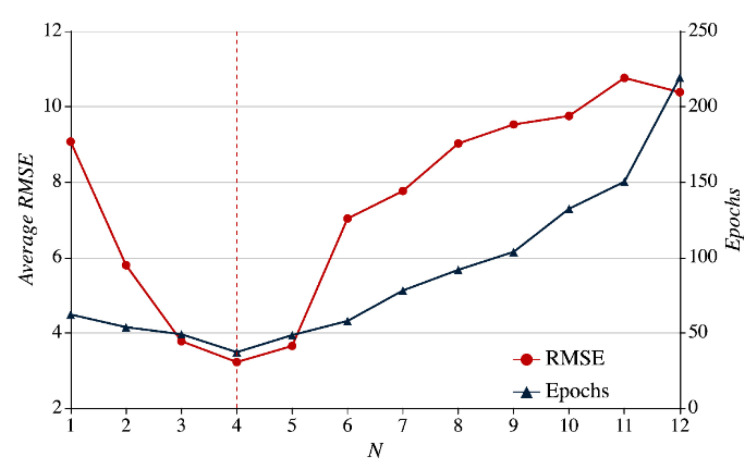
Impact on SAM error and convergence epochs with different number of sequences.

When SAM is used for prediction and assessment, the number of correlation sequence N will affect the prediction accuracy rate and the number of convergence epochs. In the prediction of 31 nodes available within the single region $r_{1}$ from 1 January 2017 to 31 December 2017 we increase the number of correlation sequence N from “1” to “12,” and make statistics of each node’s average RMSE and average convergence epochs. The experiment results are shown in Figure 14 where the horizontal axis represents the number of correlation sequences, the left *y*-axis means RMSE and corresponds to the legend of the dot, and the right *y*-axis indicates the average iteration epochs and corresponds to the legend of the triangle.

As the number of correlation sequence increases, the average RMSE takes on a trend of moving up first and then down, and the number of iteration epochs goes up as a whole except for a slight drop when there are less than four correlation sequence. Multiple correlation sequences (N≤4 as shown in Figure 14) introduce additional information, compared with a single sequence. This can not only help reduce single node prediction error but the number of model convergence epochs. However, when there are too many correlation sequences (N≤4 as shown in Figure 14), irrelevant or disrupting information may exist among these sequences to result in more error. Furthermore, the input scale-up will lead to more convergence epochs. As indicated in Figure 14, when the number of correlation sequences is “4,” both the RMSE and iteration epoch curves are found in their trough. Therefore, in the subsequent experiments, we will select four correlation sequences (N=4) to conduct single node prediction and assessment for the single region $r_{1}$. In other words, the sequence of the first three nodes related to the target node will be chosen as inputs.

(2) Correlation Coefficient Thresholds

**Figure 15:**
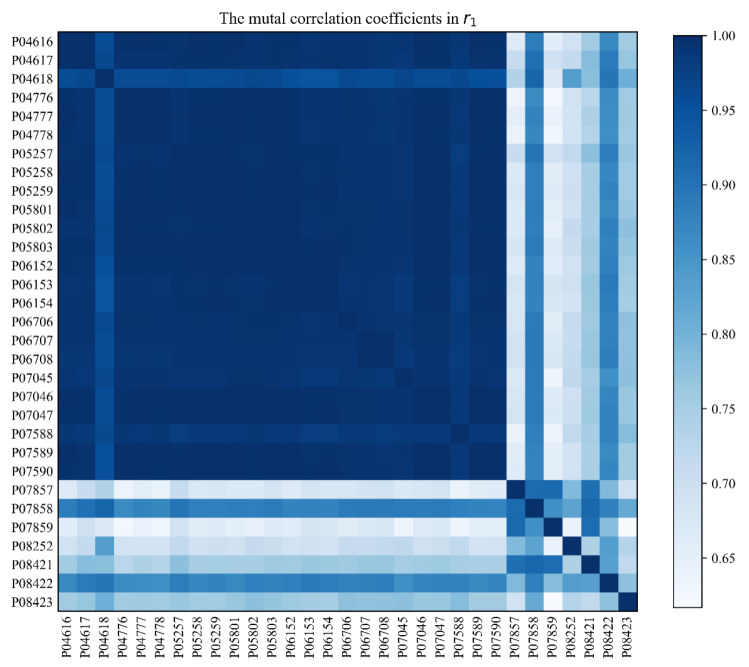
Correlation coefficients of all nodes available in single region $r_{1}$.

When the time sequence of a node bears a relatively low correlation with that of any other node, the sequence N−1 may all project a very low correlation coefficient (*Coef*) with the node, a situation which will undermine the single node prediction accuracy. The correlation coefficients of the sequence of the 31 nodes available in the local region $r_{1}$ are calculated and presented in the form of a matrix as shown in Figure 15. In the figure, both horizontal and vertical axes encompass nodes; the darker a box is, the higher the two-node sequence’s correlation coefficient will become.

**Figure 16:**
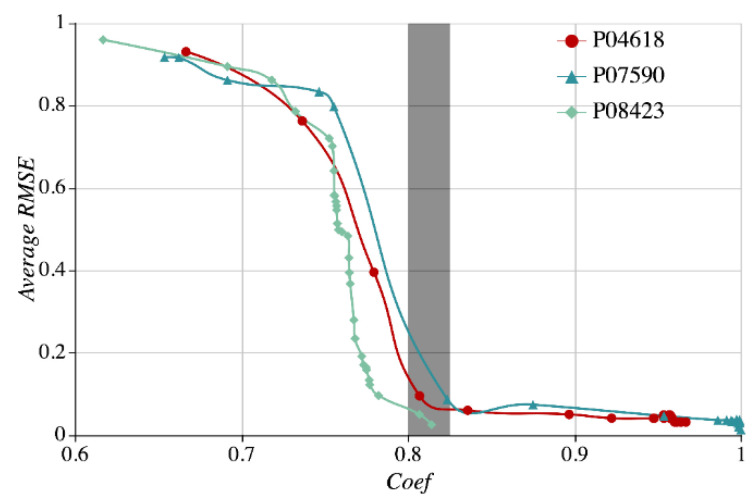
Impact on SAM error with the correlation coefficient thresholds.

Figure 16 shows that the sequence of the nodes P04618 and P07590 has a relative high correlation coefficient than that of P08423 and vice versa. With other nodes ranked by their correlation coefficients with P08423 in descending order, we select three correlation node sequence through the sliding window (namely, the number of correlation sequence N=4) every time to make statistics of the average RMSE as shown in Figure 16.

In Figure 16, the horizontal and vertical axes represent the correlation coefficient (*Coef*) and the average RMSE, the legends of the solid circle, triangle, and rhombus correspond to the nodes P04618, P07590, and P08423, respectively. Overall, the average RMSE curve goes down as the *Coef* increases. As displayed by the grey column in the figure, when the *Coef* threshold ranges between 0.8 and 0.825, the average RMSE will dive. Since more sequence *Coef*s are input, the single node prediction will see a more significant enhancement. However, at the time when the *Coef* threshold grows to a certain number, for instance about 0.9, the average RMSE will decrease at an increasingly flattened range. Therefore, the *Coef* threshold of 0.82 is set for the dam monitoring data set used in the experiment.

#### 5.2.4. Multiple Node Convergence Analysis with LREA

**Figure 17:**
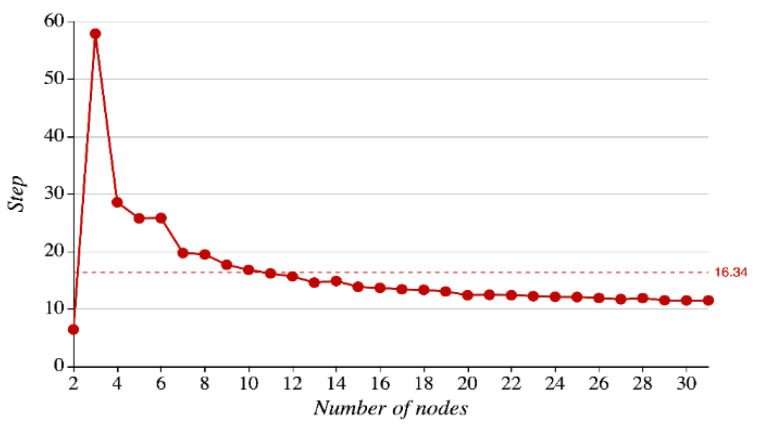
Relations between the number of nodes and the number of convergence steps.

LREA adopts the iterative solution to obtain the diagnosis results. The number of iterations affects the real-time performance of the algorithm. As preset, the number of nodes ranges between 2 and 31, the iteration threshold $\epsilon =1\times e^{-8}$, and the algorithm is run for 100 times repeatedly to make statistics of the average convergence steps as the number of nodes changes. The details can be seen in Figure 17.

In Figure 17, the horizontal and vertical axes represent the number of nodes (2–31) and the number of average convergence steps, respectively. As seen in the figure, when the number of nodes is 2, the number of average convergence steps is 6.42; when the number of nodes is 3, the number of average convergence steps will reach its peak: 57.92; when the number of nodes grows from 3 to 10, the number of average convergence steps drops rapidly, approaching the average value of 16.34; when the number of nodes exceeds 10 and continues to increase, the number of convergence steps falls at a slight and steady pace and hits its lowest level of 11.48 at the time when there are 30 or 31 nodes. In the dam safety monitoring systems, nodes usually come in a large number (more than 10), so LREA can converge fast.

**Figure 18:**
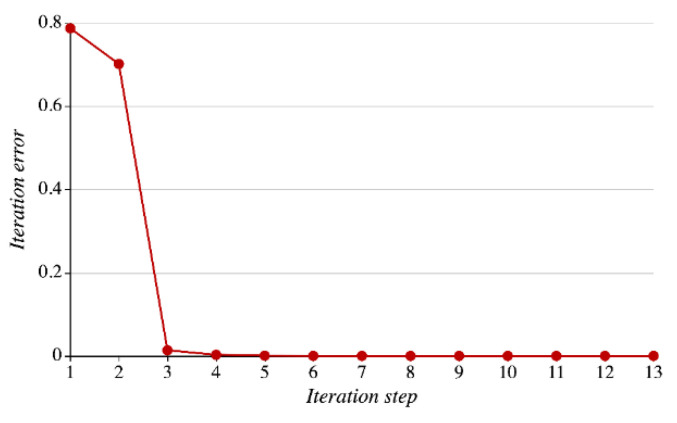
Iteration error with LREA.

Suppose that the iteration threshold is $\epsilon =1\;\times 10^{-8}$ and the number of iterations is 100. The average iteration error will change as shown in Figure 18. In the figure, the horizontal and vertical axes represent the number of iteration steps (1–13) and the iteration error, and the number of average convergence steps is 13. As shown in the figure, the iteration error plunges as the number of iteration steps increases from “1” to “3,” which evidences that LREA can get stabilized and converge rapidly.

#### 5.2.5. Local Region Evaluation Experiment with LREA, HAC, ENBC, and MMVS

(1) Impact on the accuracy

Using the 11 Key Nodes to replace the 31 nodes available in the single region $r_{1}$, we try to work out how the number of Key Nodes will impact the stability and accuracy of local region evaluation results with LREA, HAC, ENBC, and MMVS. If there are two key nodes, it needs to calculate the average accuracy rate under the $C_{11}^{2}=55$ combined schemes. *k* key nodes mean the $C_{11}^{k}$ combined schemes under which we need to make statistics of average evaluation accuracy rate.

**Figure 19:**
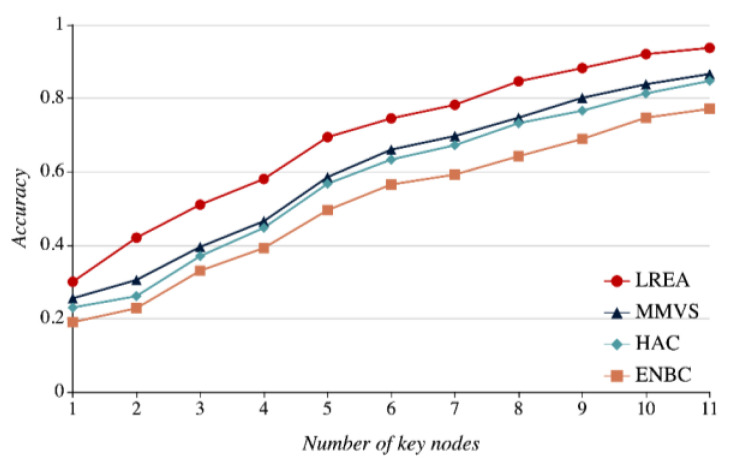
Relations between the number of key nodes and evaluation accuracy rates.

In Figure 19, the horizontal and vertical axes represent the number of key nodes and the evaluation accuracy rate, respectively. As the number of key nodes grows, local region evaluation results’ accuracy rate moves up accordingly. The four curves in the figures illustrate the evaluation results with LREA, MMVS, HAC, and ENBC from bottom to top. The LREA curve stays above the three other curves all the time. This is because the introduction of node credibility makes it possible for a single node evaluation error to be corrected by other correlation nodes. Among the three traditional methods, MMVS achieves the optimal values, thus functioning better than HAC and ENBC. Failing to consider the assessment error of different nodes, HAC and ENBC perform worse than the two other assessment accuracy methods.

(2) Accuracy Analysis of LREA, HAC, ENBC, and MMVS

**Figure 20:**
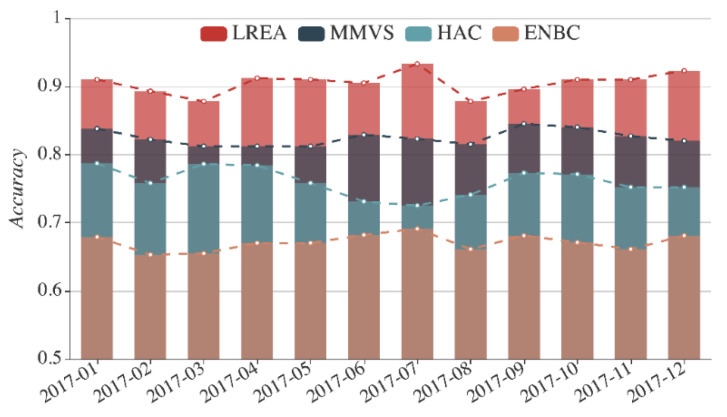
Monthly average evaluation accuracy rates of region $r_{1}$ in 2017 with the four methods.

**Figure 21:**
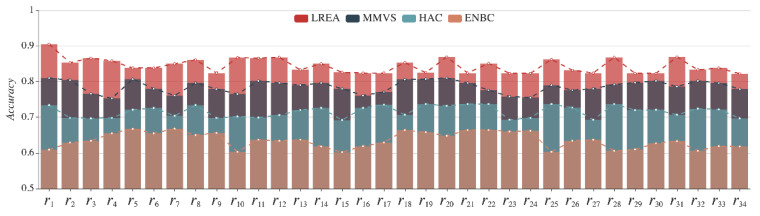
Evaluation accuracy rates of 34 local regions in 2017 with the four methods.

With the real data on the dam under monitoring from 1 January 2017 to 31 December 2017, we compare LREA with the currently used three methods: HAC, ENBC, and MMVS. The monthly average evaluation accuracy rates of the local region $r_{1}$ attained with the four methods are illustrated in Figure 20, and the annual average evaluation accuracy rates of the 34 local regions ($r_{1}$ to $r_{34}$) with the four methods can be seen in Figure 21.

In Figure 20, the horizontal axis encompasses different months, the vertical axis indicates the monthly average evaluation accuracy rates, the four tops of each colored column represent the accuracy rates of the four methods. Taking July 2017 as an example, we can see that the four columns in different colors from high to low correspond to LREA, MMVS, HAC, and ENBC, with the accuracy rates standing at 93.3%, 82.3%, 72.5%, and 69.1%, respectively. Overall, the four methods are ranked by the accuracy rates in descending order: LREA, MMVS, HAC, and ENBC. The annual accuracy rates of evaluation results obtained with LREA, HAC, ENBC, and MMVS come at 90.5%, 82.5%, 76.0%, and 67.1%.

Figure 21 shows that the horizontal and vertical axes represent the single region and the annual evaluation accuracy rates, respectively. All local regions under monitoring see their average evaluation accuracy rates obtained with LREA, MMVS, HAC, and ENBC standing at 84.2%, 78.6%, 71.6%, and 63.5%, respectively. From Figure 19 and Figure 20, it is obvious that LREA is superior to MMVS, HAC, and ENBC in terms of evaluation accuracy rate.

(3) The application system of the evaluation of the dam operation state

**Figure 22:**
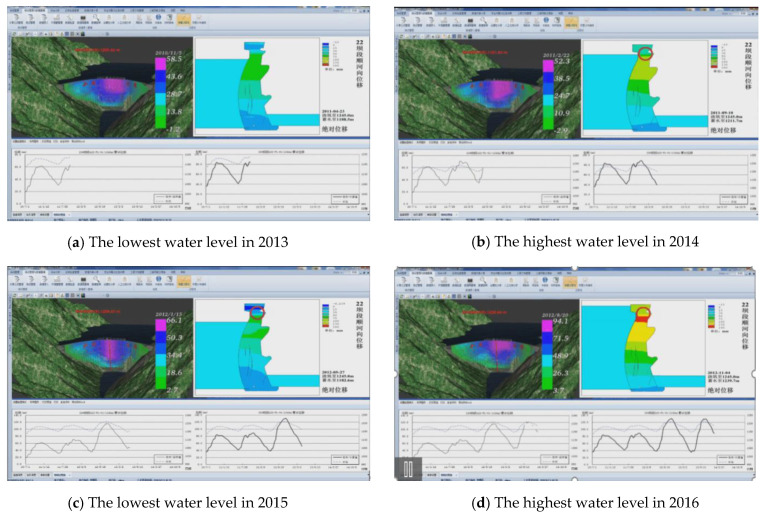
The application system for evaluation (**a**) the lowest water level in 2013; (**b**) the highest water level in 2014; (**c**) the lowest water level in 2015; (**d**) the highest water level in 2016.

To further illustrate the evaluation results of the dam operation state, we have developed and implemented an application system for the evaluation the dam operation state, as shown in Figure 22. Figure 22a,c show the evaluation results under the situation of the lowest water level in 2013 and 2015, respectively. Figure 22b,d show the results in the highest water level in 2014 and 2016, respectively. From Figure 22a–d, they illustrate the different evaluation results of the central section of the dam from the lowest water level to the highest water level when the measured values continue to change over time.

### 5.3. Experiment Conclusions

In SAM, multiple sequence data are used to complete single node prediction and assessment, thus substantially avoiding the prediction and assessment error due to missing and abnormal values in a single node. From the results of Experiment 1, we can find that SAM can promptly reflect the fluctuations in the node sequence, which enables it to outperform CM in the prediction performance. In Experiment 2, SAM and CM generate an overall assessment accuracy rate at 70.0% and 37.6%, respectively. This is an illustration that SAM out-competes CM in terms of assessment stability and accuracy. Experiment 3 is designed to analyze how the number of correlation sequence N and the correlation coefficient threshold *Coef* impacts the SAM performance. According to the measured data set of the dam under monitoring, we assume N=3 and Coef=0.82 in the experiment.

A node credibility model is created with LREA, so that multiple nodes can be coordinated to evaluate the operation state of a local region through variations in credibility. Experiment 4 aims to verify the impact on the number of convergence steps with LREA brought by the different number of nodes. The experimental results indicate that LREA can converge rapidly when there are more nodes (more than 10). Compared with three methods: HAC, ENBC, and MMVS, LREA can outperform the other three methods in terms of the monthly average evaluation accuracy rates of the local region.

## 6. Conclusions

To provide the accuracy and real-time evaluation for the dam safety state, this paper addressed the region evaluation with multiple relevant time sequences from the nodes deployed for dam safety monitoring. In this paper, we consider the correlation of data on nodes in the entire monitoring network. To avoid the deviation caused by noise and missing value in the single-node data sequence, we calculate the correlation between the multiple sequences. A single-node assessment model based on multiple relevant sequence (SAM) is proposed to improve the accuracy of single node assessment. Given the different nodes of a local region have varying impacts on the evaluation results, a local region evaluation algorithm based on node credibility (LREA) is presented to model the credibility of the nodes in order to alleviate inconsistent evaluation results in the local region of the dam. LREA can assess the dam’s operation state by considering the variations in credibility and multiple nodes coordination. The experimental results illustrate the LREA can reveal the trends of the monitoring values change in a timely and accurate way, which can elevate the accuracy of evaluation results of dam safety.

The measured values of nodes in the dam safety monitoring systems are affected by many environmental factors, such as the water level, temperature. In the future work, the evaluation of single node state can consider more influencing factors. Moreover, we will study in depth the changing law of the reliability of measurement points at different times, and optimize the iterative process of evaluation.

## Figures and Tables

**Table 1:** Dam safety monitoring dataset descriptions.

Category	Intra-Category Nodes	Key Node	Category	Intra-Category Nodes	Key Node
C1	P04616, P04617, P04618	P04618	C7	P07045, P07046, P07047	P07045
C2	P04776, P04777, P04778	P04776	C8	P07588, P07589, P07590	P07588
C3	P05257, P05258, P05259	P05257	C9	P07857, P07858, P07859	P07857
C4	P05801, P05802, P05803	P05801	C10	P08252	P08252
C5	P06152, P06153, P06154	P06152	C11	P08421, P08422, P08423	P08421
C6	P06706, P06707, P06708	P06706			

**Table 2:** Single region evaluation instance on September 12, 2017.

Key Node	Single Node Assessment Results	The Operation State of Single Nodes	Node Credibility	The Local Region Evaluation Result
P07588	[0.330, 0.284, 0.063, 0.323]	0	1.879	$d^{1}=\left[\begin{array}{l} 0.192,\\ 0.232,\\ 0.320,\\ 0.256 \end{array}\right]$ Predicted evaluation result: 2 Actual evaluation result: 2
P04776	[0.004, 0.479, 0.285, 0.232]	1	1.814	
P05257	[0.201, 0.312, 0.214, 0.274]	1	3.511	
P06152	[0.137, 0.374, 0.301, 0.189]	1	3.068	
P07857	[0.222, 0.377, 0.322, 0.078]	1	2.423	
P04618	[0.272, 0.240, 0.304, 0.184]	2	3.936	
P06706	[0.008, 0.156, 0.439, 0.397]	2	2.098	
P07045	[0.211, 0.111, 0.415, 0.263]	2	3.219	
P08421	[0.223, 0.079, 0.492, 0.207]	2	2.370	
P05801	[0.311, 0.071, 0.302, 0.316]	3	2.619	
P08252	[0.071, 0.057, 0.385, 0.486]	3	1.770	

## Data Availability

Not applicable.
